# Emergency embolization for breast pseudoaneurysm following vacuum-assisted biopsy: a case report

**DOI:** 10.11604/pamj.2021.38.135.27619

**Published:** 2021-02-08

**Authors:** Nguyen Thai Binh, Nguyen Minh Duc, Thieu-Thi Tra My, Le Viet Dung, Doan Tien Luu, Le Tuan Linh

**Affiliations:** 1 Department of Radiology, Ha Noi Medical University Hospital, Ha Noi, Vietnam,; 2 Department of Radiology, Ha Noi Medical University, Ha Noi, Vietnam,; 3 Department of Radiology, Pham Ngoc Thach University of Medicine, Ho Chi Minh City, Vietnam,; 4 Department of Radiology, Children´s Hospital 2, Ho Chi Minh City, Vietnam

**Keywords:** Vacuum-assisted biopsy, pseudoaneurysm, embolization, case report

## Abstract

Vacuum-assisted breast biopsy (VABB) is a minimally invasive procedure and has become an important treatment method. Although VABB is a minimally invasive procedure, it might cause complications, particularly those associated with blood vessels. In this article, we aimed to describe a 35-year-old female who experienced pseudoaneurysm post-VABB and was successfully treated with embolization. She presented to the hospital with a suspected left breast tumor. The pathology report after biopsy confirmed fibroadenoma, and the patient underwent VABB to remove the tumor. One hour after VABB, the patient described pain and swelling at the location of the removed tumor. Breast ultrasound revealed a hematoma and pseudoaneurysm. The bleeding did not stop following the application of manual compression. Breast hemorrhage was controlled by endovascular embolization. Pseudoaneurysm is an uncommon complication of VABB, and embolization represents an effective method for the management of breast pseudoaneurysm.

## Introduction

The introduction of breast cancer screening programs has resulted in the increased detection of benign breast tumors [1]. The definitive management of these benign lesions is preferred by patients [2]. Minimally invasive techniques for the treatment of breast lesions have been found to be effective, safe, and associated with acceptable esthetic outcomes [2]. The vacuum-assisted breast biopsy (VABB) technique can be used for both biopsy and benign tumor removal procedures [3]. VABB is performed under ultrasound guidance and typically lasts from 13-60 minutes [2]. Hematoma and bleeding are the most common complications associated with VABB [4]. The open surgical repair of a hematoma or pseudoaneurysm related to VABB is not necessary in all cases, and other minimally invasive methods, such as manual pressure, or percutaneous embolization, are increasingly utilized [5]. In this report, we present a case of VABB associated with vascular complications that were successfully treated with emergency embolization.

## Patient and observation

**Figure 1:**
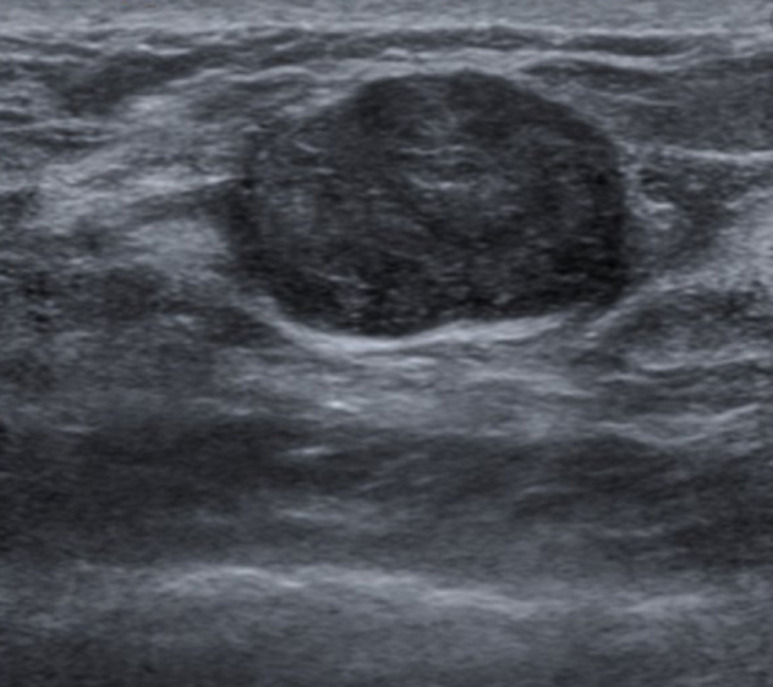
fibroadenoma at the 3 o'clock position in the left breast, measuring 20mm x 12mm

**Figure 2:**
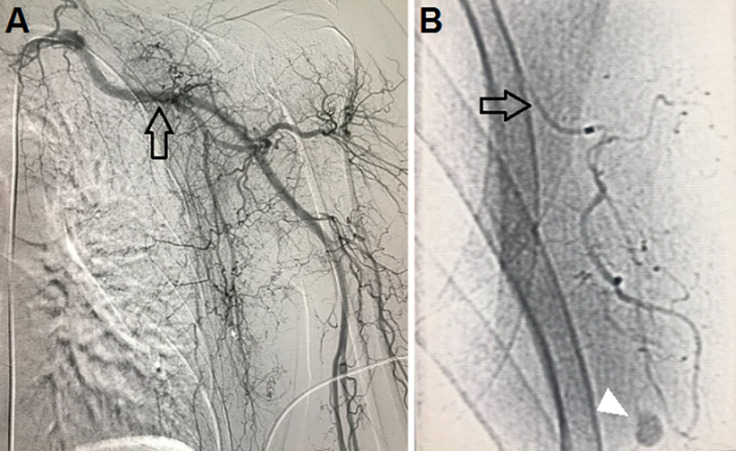
A) the left subclavian artery angiogram (arrow); B) a large pseudoaneurysm (arrowhead) in the distal branch of the left external thoracic artery (black arrow)

A 35-year-old female underwent a medical check-up due to a painless mass in the left breast, which appeared 2 years prior, without thickening or dimpling of the breast skin, pulling in of the nipple, or bloody nipple discharge. Breast ultrasound revealed a mass at the 3 o´clock position in the left breast, measuring 20mm x 12mm (Figure 1). This mass was oval and hypoechoic, with well-defined borders, oriented parallel to the skin. Pathological findings from a tumor biopsy indicated fibroadenoma. Due to a desire to treat and remove this tumor while maintaining breast esthetics and introducing minimal surgical scars, the patient underwent VABB. After the procedure, a breast ultrasound revealed no hematoma, and manual compression was performed for approximately 10 min, and the patient received a pressure dressing. One hour after VABB, the patient felt pain and swelling in the left breast. Breast ultrasound revealed a large hematoma, measuring 45mm x 30mm at the site of the prior tumor. Although the patient was treated with manual compression and injections of tranexamic acid (Cammic, Vinphaco-Viet Nam), the hematoma continued to increase in size. A pseudoaneurysm was detected on ultrasound. An intervention procedure was conducted using a 5 Fr JB2 (Terumo Corporation, Japan) catheter inserted into the left subclavian artery coronary and left axillary artery, and a selective scan of the left external thoracic artery showed a pseudoaneurysm in the distal branch of this artery (Figure 2). The pseudoaneurysm was successfully embolized using the adhesive N-butyl cyanoacrylate (NBCA) and Lipiodol (Guerbet, France), at a 1: 3 ratio. After the intervention, the patient became stabilized, and the hematoma degenerated gradually over 5 days following. A 14 Fr sonde (Bioteq, Taiwan) was inserted to drain the hematoma, and intravenous cefotaxime (Mekophar, Vietnam) was administered at 1 g every 12 hours for 3 days. After 5 days of drainage, ultrasound was used to verify that both the tumor and the hematoma were completely removed. The patient was discharged uneventfully, and at the 2-week and 3-month follow-up appointments, the patient reported no pain and minimal scarring.

## Discussion

The VABB technique, which was developed in late 1995, uses ultrasound guidance to remove samples of breast tissue through a single, small skin incision [6]. Because VABB uses a suction system, a single insertion may be used to obtain larger tissue samples for accurate diagnoses [3]. Additionally, VABB can be used for the treatment and complete removal of benign breast lesions [7]. Complications associated with this procedure may include hematoma; breast vessel injury, which can induce bleeding or pseudoaneurysm; bruises; nipple sensation abnormalities; and pneumothorax, which occurs ata mean rate of 2.5% [7-9]. Most complications are mild to moderate, and severe complications are rare [7] Zheng *et al*. [10] reported an incidence of hematoma and bleeding after VABB of 11.4% Simon *et al*. [11] reported that 7% of patients experienced bleeding that could not be controlled by manual compression within 10 minutes post-procedure. A breast pseudoaneurysm is rare, and the first two cases of transcatheter pseudoaneurysm embolization following VABB were reported by Fischman *et al*. [12]. Following this report, the application of manual compression and pressure bandage was recommended for 48-72 hours following VABB to minimize hematoma occurrence [8]. Multiple factors can contribute to the induction of hematoma following VABB, including nodule size, nodule number, breast shape, menstrual cycle stage, and the efficacy time of bandage less than 12 hours after the operation [13].

Patients with more than two nodules or nodules with a maximum diameter of 25 mm or larger were found to be associated with a significantly increased risk of hematoma after VABB [13]. Hematoma symptoms may occur immediately and present as pulsatile bleeding after VABB [5]. Pseudoaneurysm should be suspected when a patient presents with a rapidly growing breast mass at the previous tumor site after VABB [9]. Cutaneous bruising surrounding the mass may occur [9]. Ultrasound is the most common imaging modality used for the diagnosis of pseudoaneurysm [5]. Grayscale can be used to identify a new cystic mass at the biopsy site, and color Doppler imaging can be used to detect the presence of a swirling flow in the mass [14]. A pseudoaneurysm can also be identified using angiography and computed tomography scans [9]. Breast pseudoaneurysm treatment can be performed using multiple methods, including observation with external pressure dressing; ultrasound-guided, focused compression; thrombin injections; intravascular embolization; and open surgical repair [5,9]. Ultrasound-guided, focused compression is the first-line treatment option and is typically performed using 20 to 30 minutes of direct compression for the treatment of small breast pseudoaneurysms and hematomas [5]. The intravascular embolization of a pseudoaneurysm, using gelfoam, adhesives, or metallic coils, is recommended in cases with significant hemorrhage [9]. Open surgical repair is performed as a last resort when other methods fail [5]. In this case, a complication occurred, despite the tumor not being large. Due to the failure of focused compressions, embolization was required. After successful embolization, the draining of the hematoma facilitated wound healing and reduced infection risk.

## Conclusion

This case represents an incident of failure to stop VABB-induced bleeding using focused compression. Although VABB is a safe procedure, associated with a low rate of typically mild to moderate complications, breast vessel injury is always a risk. Patients who present with increasing pain and rapid growth at the tumor site after biopsy must be evaluated for possible pseudoaneurysm. This patient received a prompt diagnosis and was treated successfully with intravascular embolization.
